# The Development of a Chinese Healthy Eating Index and Its Application in the General Population

**DOI:** 10.3390/nu9090977

**Published:** 2017-09-05

**Authors:** Ya-Qun Yuan, Fan Li, Rui-Hua Dong, Jing-Si Chen, Geng-Sheng He, Shu-Guang Li, Bo Chen

**Affiliations:** Key Laboratory of Public Health Safety of Ministry of Education, Collaborative Innovation Center of Social Risks Governance in Health, School of Public Health, Fudan University, Shanghai 200032, China; yqyuan16@fudan.edu.cn (Y.-Q.Y.); 16211020023@fudan.edu.cn (F.L.); 15111020019@fudan.edu.cn (R.-H.D.); 15211020022@fudan.edu.cn (J.-S.C.); gshe@shmu.edu.cn (G.-S.H.); leeshuguang@fudan.edu.cn (S.-G.L.)

**Keywords:** dietary index, diet quality, general population, sociodemographic determinants, China

## Abstract

The objective of this study was to develop a Chinese Healthy Eating Index (CHEI) based on the updated Dietary Guidelines for Chinese (DGC-2016) and to apply it in the 2011 China Health and Nutrition Survey (CHNS-2011) to assess diet quality and its association with typical sociodemographic/economic factors. Data from 14,584 participants (≥2 years) from the CHNS-2011, including three 24-h dietary recalls and additional variables, were used to develop the CHEI. The standard portion size was applied to quantify food consumption. The CHEI was designed as a continuous scoring system, comprising 17 components; the maximum total score is 100. The mean, 1st and 99th percentiles of the CHEI score were 52.4, 27.6 and 78.3, respectively. Young and middle-aged adults scored better than the elderly. Diet insufficiency was chiefly manifested in fruits, dairy, whole grains and poultry; diet excess was mainly reflected in red meat, cooking oils and sodium. The CHEI was positively associated with education and urbanization levels; current smokers and unmarried people obtained relative low CHEI scores. Occupation and body mass index (BMI) were also related to the CHEI. Our findings indicate that the CHEI is capable of recognizing differences in diet quality among the Chinese, and it is sensitive to typical sociodemographic/economic factors.

## 1. Introduction

Good diet is essential to a healthy lifestyle. The diversity and complexity of human diets have spawned dietary pattern analysis involving a series of methods to assess diets comprehensively. With these approaches, the interactions and synergies of food groups have been covered, and the causal links between diets and chronic diseases have been clarified [1,2]. Among these methods, diet indices, as the representation of a priori dietary patterns, are characterized by assessing overall diet quality in terms of conformance to the dietary guidelines or nutrition recommendations. In addition to measuring overall diet quality, the indices have been applied in population monitoring of nutritional status, food environment evaluation, effect estimation of nutritional intervention and in epidemiology research to explore the association between diet behaviors and certain health-related outcomes [3,4,5,6,7].

A variety of diet indices have emerged in the last two decades, including the Healthy Eating Index (HEI), the Diet Quality Index (DQI), the Mediterranean Diet Score (MDS), the Healthy Diet Indicator (HDI) [8], and so forth. Among these indices, the HEI is the most well known. The HEI was initially established in 1995 to measure the diet quality adherence to the Dietary Guidelines for Americans (DGA), and it was revised twice along with the release of the DGA-2005 and DGA-2010 [9,10]. Compared with other diet indices, the HEI series have several methodological advantages. Firstly, they were designed as a continuous scoring system (as opposed to discrete), which made them easy to interpret and to allow for diverse statistical analyses. Secondly, the HEI-2005 and HEI-2010 adopted a density standard, proving a common criterion for different subpopulations, which avoided the problem of calculating an individual’s energy requirement [9,10]. Additionally, the validity and reliability of the HEI-2005 and HEI-2010 have been examined explicitly [11]. These advantages effectively accelerated their promotion worldwide. Canada, Brazil, Australia and Thailand have adapted the HEI for their own populations based on local dietary guidelines and nutritional status [12,13,14,15]. The HEI-2005 and HEI-2010 and versions adapted for different countries have been used to assess diet quality and the associations between the diets and sociodemographic/economic indicators, chronic disease (e.g., cardiovascular disease, type 2 diabetes, cancer) and mortality [4,5,7].

In China, three diet indices have been developed based on the Dietary Guidelines for Chinese (DGC) and the Chinese Food Pagoda (CFP): the Chinese Dietary Balance Index (DBI), the DQI for China and Chinese Food Pagoda Score (CFPS). The DQI and DBI were similar in bidirectional scoring system. Excessive consumption and inadequate intake were assessed by the high bound score (>0) and the low bound score (<0), respectively [16,17]. The CFPS was developed as a discrete scoring system and ranged from zero to ten [18]. All three indices are effective measures of diet quality and have been applied in different populations of China [17,18,19]. However, studies of the relationship between diet quality and diseases are still rare in China, which is possibly because that the bidirectional or discrete scoring method of existing indices limited their application in statistical analysis and further in diet-disease research.

In recent decades, rapid economic progress and profound changes in lifestyle have brought an increasing prevalence of multiple chronic diseases in China. Among Chinese adults, the prevalence of obesity increased from 7.1% in 2002 to 11.9% in 2012; the prevalence of hypertension increased from 18.8% to 25.2% during the same 10 years [20] (pp. 90–130). Almost half of the elderly suffered from hypertension in China [20] (pp. 90–130). The prevalence of diabetes in China has more than quadrupled in recent decades, with an estimated number of 110 and 490 million adults having diabetes and prediabetes, respectively, in 2010 [21]. In the meantime, an unbalanced diet resulting from the nutrition transition has been widespread across China. Thus far, existing literature has primarily focused on the relationship between certain chronic diseases and unhealthy diet behaviors based on single food or nutrient measurement, including high sodium consumption, low fruit intake [22], and so forth. However, the causal links between chronic diseases and overall diet quality in China remain unclear.

The most recent Dietary Guidelines for Chinese (DGC-2016) was released in 2016. In addition to performing several alterations on food groups and intakes, the DGC-2016 has brought scientific evidence to support the key recommendations. In view of the limitations of existing diet indices in China and the methodological advantages of the HEI, we aimed to develop a new Chinese Healthy Eating Index (CHEI) based on the DGC-2016. With CHEI, we also aimed to assess the diet quality for participants of the China Health and Nutrition Survey (CHNS)-2011 and the associations between the diets and typical sociodemographic and socioeconomic indicators.

## 2. Materials and Methods

### 2.1. The DGC-2016 and the Standard Portion (SP) Size

The Dietary Guidelines for Chinese is the foundation of nutrition policy and the basic document of public health care and education in China. The dietary guidelines were originally released in 1989 and revised nearly every 10 years by the Chinese Nutrition Society. The DGC-2016 are the most current and scientific nutritional guidelines for the Chinese. The most pronounced feature of the current edition is that it is evidence-based, which indicates that each key recommendation of DGC-2016 was supported by sufficient health-related evidence.

In addition to revising recommended food intakes, the DGC-2016 proposed the concept of a standard portion (SP) for food quantification. In China, grams (g) or kilograms (kg) are prevalent units for food measurement both in academic research and daily life. They measure food intakes based on the absolute amount of weight, which might result in ambiguity. For instance, 100 g rice and corn are equal in weight, but they are different in energy and nutrient contents. To manage this obscurity and to quantify food groups with unity, the standard portion (SP) was proposed to help the public meet their nutritional needs. Based on the DGC-2016, identifying SP size for food groups should abide by the principle of consistency in energy and protein and conformity in dietary custom of Chinese people. One SP size of foods in one food group should share consistent content of energy and similar contents of carbohydrates, fats and protein. Using the database of the China Food Composition, we calculated the specific contents of energy, carbohydrates, fats and protein in one SP size of each food category (Supplemental Table S1). In our analysis, a total of 1289 kinds of single food collected in g are divided into 10 groups, additionally converted into the SP size for development of the CHEI. Oil, added sugars, alcohol and sodium are estimated in g.

### 2.2. Participants and Dietary Assessment

The present study used the data of the China Health and Nutrition Survey (CHNS). The CHNS is a prospective, ongoing open cohort study conducted since 1989, drawing samples using a multistage, random cluster method. Four major regions (Northeast China: Heilongjiang, Liaoning; East Coast: Shandong, Jiangsu; Central China: Henan, Hubei, Hunan; and the West: Guangxi, Guizhou) have been involved in the CHNS since 2000, which mainly accommodated all levels of socioeconomic development across China. Details of the CHNS are described elsewhere [23].

In the present study, data from 24-h recalls over three consecutive days at an individual level were used for analysis. Additionally, oil and sodium intakes from household food inventory data over the same three-day period were used to supplement the individual dietary data. At the individual level, participants reported all foods consumed at home and away from home in a 24-h period. Trained interviewers recorded types, amounts and the eating location of all foods at each meal, using food models and pictures. Household food consumption was estimated by examining the daily changes of inventory, using a weighing and measuring technique as described in detail elsewhere [24]. Data from 14,584 participants of the 2011 survey were used in the present study. Children under two years of age were excluded because the DGC-2016 is designed to meet the needs of people two years and older.

### 2.3. Relevant Variables and Statistical Analysis

Education level was divided into three categories from six initial levels in the questionnaire: low (primary school and lower); medium (junior and senior middle school), and; high (college middle school and higher). Smoking status was identified as non-smokers, former smokers, and current smokers. Marital status was expressed as living alone (never married, divorced, widowed, and separated) or not (married). Urbanization was determined as rural, suburban or urban according to the information from the questionnaire. Occupational status consisted of five categories: the employed; the unemployed (of working age); students; school dropouts, and; the retired. Body mass index (BMI) values were divided into four categorical levels based on the Working Group on Obesity in China: underweight, BMI < 18.5 kg/m^2^; normal, BMI 18.5–23.9 kg/m^2^; overweight, BMI 24.0–27.9 kg/m^2^, and; general obesity, BMI ≥ 28 kg/m^2^.

Total CHEI score was expressed with mean and standard deviation (SD) as it was normally distributed. The Cochran-Mantel-Haensel (CMH) test was used to assess the correlation between tertiles of CHEI score and the characteristics. Scores of each of the 17 CHEI components were divided into four sections, which were 0, (0–2.5), (2.5–5), 5 for all components except fruits, sodium and cooking oils, and 0, (0–5), (5–10), 10 for fruits, sodium and cooking oils. Differences in sections of the CHEI components score between sex and age groups were estimated by the chi-square test. Associations between the total CHEI score and associated factors were explored by both univariate and multivariable linear regression models. The statistical analysis was performed by SAS software version 9.3 for Windows (SAS Institute Inc., Cary, NC, USA). Two-side *p*-values < 0.05 were considered statistically significant.

## 3. Results

### 3.1. Development of the Chinese Healthy Eating Index

#### 3.1.1. Components

The development of the Chinese Healthy Eating Index was based on three crucial foundations: key recommendations in the DGC-2016; current dietary status across China (Supplemental Figure S1a,b); and the evidenced association of identified components with relative health outcomes. Seventeen components mapped to the key dietary recommendations of the DGC-2016 are determined (Table 1), assessing diet quality from two angles: adequacy (total grains, whole grains and mixed beans, tubers, total vegetables, dark vegetables, fruits, dairy, soybeans, fish and seafood, poultry, eggs, and seeds and nuts), and limitation (red meat, cooking oil, sodium, added sugar and alcohol). Only food groups proved to be associated with certain health-related outcomes are incorporated in the CHEI (Table 1).

The DGC-2016 recommends consuming a cereal-based diet, especially increasing the intake of whole grains, tubers and mixed beans, which can all be regarded as staple foods. Since the whole grains and mixed beans are categorized into one group and share the combined recommended intake amounts in the DGC-2016, they are also considered as one component in the CHEI. Cereals, rather than animal foods, should primarily provide dietary energy (50–65%). Healthy diets necessitate a sufficient and diverse intake of fresh vegetables and fruits. The DGC-2016 recommends consuming vegetables at every meal, and consuming dark vegetables at least in half of them. Fruits are needed every day, and should be in nutrient-dense forms. Based on the DGC-2016, dairy is necessary for a healthy diet. A variety of dairy products, including milk, yogurt, cheese, and milk powder are available. Soybeans and other products rich in plant protein should be consumed regularly. Seeds and nuts are good for health because they are rich in various unsaturated fatty acids, vitamins and minerals. However, over consumption will lead to an excess of energy. In the present study, seeds and nuts are regarded as an adequacy component since they are widely inadequately consumed among Chinese.

For a healthy diet, various sources of protein food including fish, seafood, meat, poultry and eggs are all needed since they are rich in different kinds of nutrients. However, the proportion of protein foods consumed by the Chinese is unbalanced. Excessive intakes of red meat and inadequate consumption of poultry, fish and seafood are coexistent. Considering the current dietary status, the DGC-2016 recommended increasing intakes of fish, seafood, and poultry and moderately consuming red meat.

High consumption of cooking oils and salt is a conspicuous feature of Chinese dietary habits. Although cooking oils are rich in essential fatty acids and vitamin E, excessive intakes of any kind of fat will increase energy intake and the risk of obesity. Based on the CHNS, the sodium in salt accounts for 72% of the total sodium intake. In addition to sodium from salt, the sodium component also includes sodium from sodium-rich foods (sodium content ≥ 500 mg/100 g), including soy sauce and other condiments. Due to the status of their over consumption across China, the DGC-2016 recommends consuming cooking oils and sodium within certain limits.

Added sugars include all sugars used as ingredients in processed or prepared foods, such as breads, cakes, soft drinks, jams, chocolates, and ice cream, and sugars eaten separately or added to foods at the table. When added to foods or drinks, sugars provide calories without necessary nutrients, which makes it difficult for individuals to meet their nutritional needs within appropriate caloric limits. The DGC-2016 recommends consuming added sugars within certain limits (Table 1). Since no data on added sugars is in Chinese Food Composition database, the component is calculated using the USDA database [26]. Alcohol drinking, especially long-term drinking, is related to various adverse health outcomes. According to the Chinese Report of Nutrition and Chronic Disease 2015, the adults' harmful drinking rate had reached 9.3% (men 11.1%, and women 2.0%). Therefore, alcohol is a limitation component in CHEI.

#### 3.1.2. Weighting

The CHEI has a continuous scoring system; the maximum total score is 100. Firstly, 17 components were weighted identically by assigning a maximum of five points each, supposing that they were equally important, which reflected the instruction in DGC-2016 that all recommended food groups are needed, and no one could be replaced by others. Then cooking oils, sodium, and fruits were weighted twice as heavily for the following reasons. Excessive intake of cooking oils and sodium is a long-term feature of Chinese dietary habits, which has been linked with various adverse health outcomes. Additionally, as condiments, they are consumed by nearly everyone at each meal. Compared with other food groups, long-term over intake of cooking oils and sodium will have a more profound and lasting impact on health. Therefore, cooking oils and sodium are both assigned 10 points. Fruits are considered equally important as vegetables in their contributions to a healthy diet and are also allotted 10 points. The maximum scores of the two vegetable components also sum to 10.

#### 3.1.3. Scoring

Scoring for the CHEI components is based on the energy density (as amounts per 1000 calories of intake). In DGC-2016, the key recommendations of each food group are converted into specific and quantified amounts at 12 caloric levels (Supplemental Table S2a,b). Recommended amounts for each food group-based component are converted and expressed in SP/1000 kcal, and cooking oils is expressed in gram/1000 kcal (Table 2). For all food groups listed in Table 2, the 1600–2400 caloric patterns were selected to determine the cutoff points because they were used to set the general recommended amounts of the DGC-2016. Scoring for the CHEI adopted the least-restrictive approach, which indicated that among the 1600–2400 caloric patterns, the least-restrictive standards were set as the cutoff points for the maximum score (Table 3).

Adequacy components specified food groups that were consumed deficiently across China, needing an increase in intake. For the 12 adequacy components, the lowest recommended amounts in 1600–2400 calorie interval, expressed in SP/1000 kcal, were identified as the cutoff points for the maximum score. Intakes at the level of the cutoff points or better received the highest score. No intakes received a score of zero.

Limitation components specified food groups that should be limited or consumed in moderation. For these components, the cutoff points for the maximum score were the highest recommended amounts in 1600–2400 calorie interval, expressed in SP/1000 kcal. Meanwhile, a cutoff point should be designated to specify how high an intake deserves a zero score. Referring to the experience of the HEI-2010, the cutoff for a zero score should ensure that there is not a large percentage of people who get the zero score. Based on the distribution of three-day intakes of the participants, an estimate of the 90th percentile of the population is identified as the cutoff point for a zero score.

Based on the Chinese Dietary Reference Intakes (DRIs), the adequate intake (AI) for sodium is less than 1500 mg/day. Studies from the DASH (Dietary Approach to Stop Hypertension) indicated that a diet containing 1500 mg sodium is beneficial in preventing hypertension without the probability of deficiency [27]. Additionally, the Chinese DRIs also recommended 2000 mg/day for sodium as the Proposed Intakes (PI) for Preventing Non-Communicable Chronic Diseases for adults [28]. Since the development of the CHEI is based on the least restrictive approach, 2000 mg/day is determined as the standard for the maximum score of 10. For estimating sodium intakes in an energy density form, the 2000 kcal caloric level was selected to calculate the cutoff points because it is the median of the total energy levels involved in the DGC-2016. Therefore, for sodium, the cutoff points for the maximum and minimum score were 1000 mg/1000 kcal (2000 mg (PI)/2000 kcal) and 3608 mg/1000 kcal (90th percentile), respectively.

In the DGC-2016, the recommended intake of added sugars is less than 10% of energy intake, optimally less than 5%. Therefore, 10% of total energy intake was determined as the cutoff point for the maximum score of 5 and the 20% of energy intake (percentile 90th) is assigned as the minimum score of zero.

Drinking alcohol is not recommended by any dietary guidelines for any reason. DGC-2016 set different alcohol limitations for subgroups. For adult men and women, alcohol should be limited to less than 25 g/day and 15 g/day, respectively. Children, adolescents (under 18 years old), pregnant and lactating women should not drink alcohol. Based on the World Health Organization (WHO)’s Global Alcohol and Health Report, 60 g/day and 40 g/day are defined as harmful drinking amounts for men and women. Accordingly, different standards are set for different subgroups. Children, adolescents, pregnant and lactating women who consumed alcohol get the score of zero, otherwise they receive the score of 5. For adults of legal drinking age, 25 g/day (men) and 15 g/day (women) are selected as the standard for the score of 5, and 60 g/day (men) and 40 g/day (women) are chosen as the standard for the score of zero.

For all the CHEI components, scores for amounts between the cutoff points for the minimum and maximum scores are prorated linearly.

### 3.2. Application of the Chinese Healthy Eating Index

The CHEI was applied for the participants of the CHNS-2011 to evaluate the overall diet quality and its associated factors. Analyses were performed on 14,584 participants who had complete dietary data and relevant covariates. Few people scored very high or very low. Only 1.8% and 5.3% of the participants had a mean scores below 30 and higher than 70, respectively. More than 60% of the participants were scored between 40 and 60. Characteristics of participants in tertiles of CHEI scores are shown in Table 4. The distribution of tertiles of the CHEI score was significantly different in participants with different characteristics of smoking status, marital status, urbanization, education level, occupation, and BMI. Estimated mean and percentiles of the total CHEI score for different age and sex groups are shown in Table 5. Total scores ranged from 27.6 at the 1st percentile to 78.3 at the 99th. Men and women had similar scores at means and percentiles. People aged 18 to 59 years tended to have higher scores at most of the percentiles of the CHEI scores when compared to the other two age groups.

**Figure 1:**
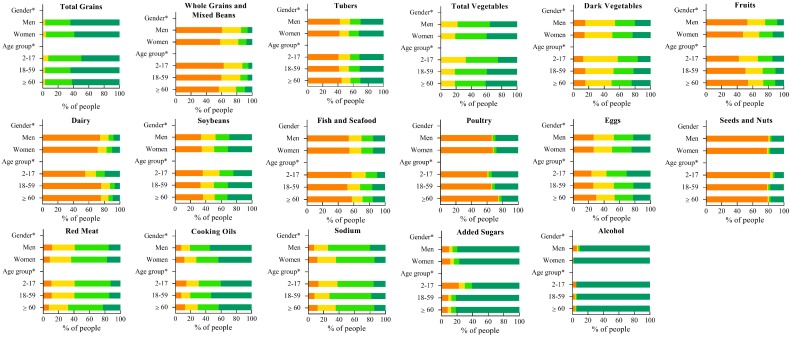
Proportion of participants in four section scores of the 17 CHEI components, according to the sex and age groups (years). For all components except fruits, sodium and cooking oils, the four sections are 0 ■, (0–2.5) ■, [2.5–5) ■, 5 ■. For fruits, sodium and cooking oils, the four sections are 0 ■, (0–5) ■, [5–10) ■, 10 ■. * Significant differences in four section scores of the 17 CHEI components between sex or age groups (χ^2^ test): *p* < 0.05.

Scores of each of the 17 CHEI components were divided into four sections, which were 0, (0–2.5), (2.5–5), 5 for all components except fruits, sodium and cooking oils, and 0, (0–5), (5–10), 10 for fruits, sodium and cooking oils. Figure 1 showed the proportion of participants in the four sections of the 17 CHEI component scores. In general, most people did not meet the recommendation (obtain the maximum component scores) of food groups in the DGC-2016, except for total grains, added sugars, and alcohol. Whole grains and mixed beans, fruits, dairy, poultry, fish and seafood, and seeds and nuts were components that had a relative serious degree of deficiency intake among the 12 adequacy components. Proportions of participants who got zero points for the six components were 59.3%, 50.2%, 72.8%, 66.7%, 53.9% and 79.0%, respectively. Meanwhile, more than half of the participants consumed more cooking oils and sodium than the recommendations. Sodium is the component that had the most serious degree of excess intake. As to added sugars, children and adolescents were more likely to consume excessively than adults and elders. The vast majority of people scored highly on alcohol consumption. The score differences in the four sections of the 17 components between sex or age groups are all significant except for fish and seafood, poultry, and seeds and nuts. Women scored better than men in components of whole grains and mixed beans, tubers, total vegetables, dark vegetables, fruits, dairy, soybeans, eggs, red meat and alcohol; while men had better scores in components of total grains, cooking oils, sodium and added sugars. For most of the CHEI components, young and middle-aged adults scored better than both juveniles and the elderly.

The relationship between the CHEI scores and the potential impact factors was assessed by linear regression models of both univariate and multivariate analysis (Table 6). The final multivariate model indicated that all listed factors were significantly associated with overall diet quality. Men and women were similar in diet quality; young and middle-aged adults had a better diet quality than the elderly. Compared with nonsmokers, current smokers had a poor diet quality. Individuals who lived alone scored lower than people who lived with others. Participants in different levels of education and urbanization showed the most pronounced discrepancies in the CHEI scores. People with high educational level had a CHEI score 5.13-fold higher than those with a lower education level. Similarly, people who lived in urban areas had 6.83-fold higher score than those in rural areas. Compared with people who were employed, the unemployed had significantly lower score, and the retired had higher score. Adolescents at school had a similar CHEI score with employed adults, while the dropouts had a relative poor score. As to BMI, people who were underweight had a lower CHEI score than people who were normal weight, but no significant difference was discovered in the CHEI scores between normal weight and overweight or obesity.

## 4. Discussion

In this study, we developed a new instrument, the CHEI, to assess the overall diet quality in terms of conformance to the DGC-2016. After building the CHEI, we described its profiles in 14,584 participants of the CHNS-2011 according to their sociodemographic/economic factors. Results showed that the overall diet quality of the participants was not satisfactory; most people scored between 40 and 60. The CHEI was capable of recognizing the differences in diet quality between sex or age groups, and was significantly associated with smoking status, marital status, urbanization, occupation, educational level, and BMI.

The DGC-2016 was the foundation for the establishment of the CHEI. The primary purpose of the CHEI is to quantify the gap between intakes and recommendations, which requires that the CHEI should capture all key aspects of the dietary guidelines. The DGC-2016 comprises six key recommendations in total, four of which reflected specific food consumptions, which are all incorporated into the CHEI. The other two, maintaining a healthy weight and stop food wasting, were rationally not included. Another purpose of the CHEI is to estimate the association between diet and chronic disease; therefore, it is important to ensure that all food groups selected as the CHEI components were linked with particular health-related outcomes (Table 1) [8]. Due to the inadequate evidence related to chronic diseases, water consumption was also not included. Diversity is necessary for a balanced diet, but whether to involve food variety as a component in a diet index is still controversial. Some researchers believe that it is superfluous to do so because it might result in overlapped scoring on other components. In the case of the CHEI, the 17 components represent different food categories. People who get non-zero scores on components would naturally receive scores on a food-variety variable, if established. Therefore, we thought there was no need to incorporate food variety as another component in the CHEI [29].

Compared with the existing Chinese diet indices, the CHEI has several advantages. Firstly, the CHEI is designed as a continuous scoring system. It is believed that consecutive scoring is more effective in capturing diet changes both in population monitoring and interventional studies than dichotomous scoring [9]. Moreover, the dichotomous approach might not give good predictions when the outcome variables are continuous rather than binary, such as biological markers. Secondly, CHEI was developed based on the density standard and the least-restrictive scoring method (Table 3). Density standards are applied because they allow a common standard to be used, and also because they have the advantage of being independent of an individual’s energy requirement, which is difficult to measure accurately and precisely. The use of the least-restrictive standards results in the error being in the same direction, which is advantageous for interpretation [10]. Thirdly, this is the first study, to our knowledge, to quantify food consumption using the standard portion sizes proposed in the DGC-2016. The application of SP will make the CHEI more effective for estimating diet quality. In the present study, the 90th percentiles of the population instead of the 85th, which were used for several indices [10,15,30], were selected as the cutoff points for limitation components. That is because, in our data set, most components showed a positive skewedness distribution, in which case the 90th percentiles would have a relative long interval from the other cutoff values, making the CHEI more informative. Additionally, it is acceptable also because the 90th percentile has a certain degree of comparability with the 85th in other indices.

The overall diet quality of the 14,584 participants was not satisfactory. The CHEI clearly revealed the existence of a high level of deficit or excessive food consumption, which has been reported to be a pronounced problem in Chinese diets. Differences in both CHEI total scores and component scores between different sex groups or age groups were detected, illustrating that the CHEI was capable of recognizing meaningful differences in diet quality between groups at one point in time.

In our data set, relatively severe diet deficiencies were found for fruits and dairy, which is accordant with the results of previous studies [18,31]. Based on the World Health Report (2014), low vegetable and fruit intake is one of the top ten risk factors contributing to mortality. Findings from the China Kadoorie Biobank indicated that among Chinese adults, higher consumption of fresh fruit was associated with significant lower mortality from cardiovascular disease (CVD), cancer, and chronic obstructive pulmonary disease [22]. The DGC-2016 also emphasized the importance of sufficient intakes of vegetables and fruits, especially dark vegetables and fresh fruits. Inadequate dairy consumption results in insufficient intake of calcium, which may lead to osteoporosis and bone fracture, especially in older people [32]. National survey data in China indicated that Ca deficiency is a serious undernutrition problem affecting people of all ages [33]. The problem of inadequate dairy intake could be due to the low per-capita supply of dairy products. It has been reported that China accounted for only 3.5% of the world’s total dairy production, which was much lower than the average of developing countries, despite its larger population [33]. In addition, the widespread lactose intolerance and lactase insufficiency among Chinese population is another drawback to insufficient consumption of dairy products. In China, for children aged 3–5, 7–8 and 11–13, the prevalence of lactase deficiency was 38.5%, 87.6%, and 87.8%; and the prevalence of lactose intolerance was 12.2%, 32.2%, and 29%, respectively [34]. The prevalence of lactose malabsorption among Chinese adolescents and adults was 76.4% to 92.3%, respectively [35]. Yogurt or low-lactose dairy can be the first choice for people with lactose intolerance.

Staple foods, including grains and tubers, are a major energy resource of a traditional dietary pattern in China. In the present study, although most participants obtained a relatively high score on total grains, nearly 60% of the participants consumed neither whole grains nor mixed beans, suggesting that large proportions of the total grains consumed were refined grains, which have been linked with various adverse health outcomes. A study in Han Chinese adults revealed that high consumption of foods with a higher dietary glycemic index (GI) or glycemic load (GL), such as refined rice and wheat, was related to a less favorable glucose homeostasis [36]. However, considering the important position of cereal in Chinese traditional diets and its reduced consumption since 1982 [37], the DGC-2016 only advised to increase the intake of whole grains and did not explicitly restrict the consumption of refined cereals. Accordingly, refined grains were not incorporated in the CHEI.

In our study, large numbers of participants consumed cooking oils and sodium moderately or severely higher than the recommendations. High-fat diets, particularly characterized by diets rich in cooking oils, would increase the risk of obesity among the Chinese [38]. Meanwhile, researchers also believe that the types of fatty acids are more important than the total amount of dietary fats, especially in increasing the risk of cardiovascular disease. Considering the nutritional status of cooking oils across China, the positive effects of the desired fatty acids cannot neutralize the adverse effects of using an excessive amount of cooking oils. Therefore, total consumption of cooking oils should be decreased, meanwhile cooking oils with a high content of beneficial fatty acids should be the preference. High sodium intake increases the risks of hypertension, stoke, and cardiovascular disease [39,40,41]. Sodium restriction has become a major measure to prevent and control hypertension around the world. However, studies have indicated that both low sodium intakes and high sodium intakes are associated with increased mortality [42], and that lower sodium intakes were not associated with lower blood pressure [43]. Nevertheless, in light of the severely excessive intake among Chinese people, sodium should be consumed within certain limits.

Alcohol consumption in our study was lower than the estimation in the Report on Monitoring of Nutrition and Health of Chinese [20] (p. 31), which perhaps because that the three-day average intakes may not represent the usual intakes, especially for foods consumed episodically, including alcohol, whole grains, fish, and seafood. Although the intake of added sugars was not too high among adults, it was high among children (Figure 1), which is worthy of attention [44]. Added sugar intake in our study might be underestimated due to the absence of relevant data in the Chinese Food Composition. To estimate the health effect of added sugars for the Chinese, especially for children and adolescents, data and information of added sugars should be incorporated in the Chinese Food Composition and labeled on products sold.

The CHEI was associated with education and urbanization levels, marital and occupation status, smoking, and BMI. Our results supported the view that high-quality diets were generally consumed by affluent and better educated people [45], and the view that individuals with low socioeconomic status (SES) were less likely to follow the guideline recommendations [46]. Researchers have found that people who were not married had a lower diet quality than people who were married [47]. A population-based study of the EPIC-Norfolk cohort found a clear and consistent reduction in both the quantity and variety of fruit and vegetable intakes (as indicators of high-quality diets) in widowed men [48]. Marriage confers individuals with a sense of obligation and meaning in life, facilitating a person's motivation to engage in healthy behaviors [49]. Occupation can impact dietary behaviors through workplace cultures and social networks [50]. Our findings indicated that the retired had a higher diet quality than others, which is consistent with previous studies [31]. The unemployed and school dropouts had a poorer diet than their opposites, which probably because that the economic pressure they face has a negative influence upon their diet behaviors.

Diet quality has been shown to be inversely associated with the intensity of tobacco consumption [51], which was supported by our results. It is plausible that individuals with better health and diet awareness never smoke. It is also conceivable that former smokers stopped smoking and tended towards good diets for health-related causes. In the present study, no significant difference was discovered in the CHEI scores between normal weight and overweight or obesity. Data collection of the three days of 24-h recalls, which cannot represent the average long-term intakes, might obscure the association between the CHEI and BMI. Further studies are needed to reveal how BMI is linked with diet quality based on the long-term intakes of food.

The strengths of the present study are as follows. This is the first instrument to evaluate the overall diet quality in accordance with the updated DGC (DGC-2016), reflecting its most current alterations. It is also the first time that the methods of standard-portion size and density standard were applied to quantify food consumption and assess diet quality among the Chinese. Although possibly underestimated, the estimation of added sugars among the Chinese is not frequent in previous literature. Additionally, sodium from sodium-rich foods was incorporated in the evaluation of total sodium intakes to address the common underestimation of diet salt. However, several limitations should be mentioned. Firstly, the CHEI cannot be applied to children under two years of age. Its application in particular populations, including pregnant and lactating women, young children and the vegetarian population needs further examination. Secondly, the CHNS is not a national survey, and the data of the CHNS do not represent national dietary status.

## 5. Conclusions

As an instrument for assessing the overall diet quality as specified by the DGC-2016, the CHEI is capable of recognizing differences in diet quality among the Chinese. The CHEI is sensitive to typical sociodemographic and socioeconomic indicators.

## Figures and Tables

**Table 1:** Components and weighting of Chinese Healthy Eating Index (CHEI) mapped to the key recommendations of the Dietary Guidelines for Chinese-2016 (DGC-2016). Grades of the evidence (ranked as A, B and C) were based on the Food and Health Evidence Based Review [25].

Components	Weighting	Key Recommendations	Comments	Key Evidence
Total Grains Whole Grains and Mixed Beans Tubers	5 5 5	Eat a variety of foods, cereal-based. Consume cereal at every meal. Increase intake of whole grains and mixed beans. Cook tubers in various forms to increase consumption.	Whole grains prevalent in China are coarse rice, whole wheat, corn, millet, buckwheat and oats. Mixed beans are rich in carbohydrates, including mung bean, red bean and kidney bean, etc. Tubers, such as potatoes, sweet potatoes and cassava, are also recommended as staple food for their high content of carbohydrates.	Consumption of whole grain reduces the risk of colorectal cancer, type 2 diabetes, cardiovascular disease and weight gain (B). Increased tuber intake reduces the risk of constipation (C).
Total Vegetables Dark Vegetables Fruits Dairy Soybeans	5 5 10 5 5	Eat plenty of vegetables, fruits, dairy products, and soybeans. Eat vegetables every meal, and half should be dark vegetables. Every day consume fresh fruits rather than processed forms. Consume a variety of dairy. Consume soybeans regularly. Seeds and nuts are beneficial, but should not be consumed excessively.	Dark vegetables include deep green, orange, red and fuchsia vegetables, such as spinach, tomatoes, carrots, and purple cabbage. Cooked, canned, frozen, and dried fruits cannot replace fresh fruits. Yogurt or low-lactose dairy products should be the first choice for individuals with lactose intolerance. Although seeds and nuts are beneficial for health, excessive intake can lead to an excess of energy.	Total vegetable consumption reduces all causes of mortality and the risk of cardiovascular diseases (CVDs) and cancers of the digestive tract; intake of dark green vegetables lowers risks of type 2 diabetes and lung cancer (B). Increased fruit intake lowers the risk of CVDs, cancers of the digestive tract and adult weight gain (B). Low-fat milk consumption decreases the risk of breast cancer, and higher dairy intake is linked to higher bone mineral density (B). Soybean intake lowers the risks of breast cancer, osteoporosis, type 2 diabetes, hyperlipidemia and hypertension (B). Moderate intake of seeds and nuts decreases all causes of mortality and the risk of CVDs, hypertension, and colorectal cancer in women (B).
Fish and Seafood Red Meat Poultry Eggs	5 5 5 5	Eat moderate amounts of fish, poultry, eggs, and lean meats. Choose fish, seafood and poultry. Decrease intake of fat meat and smoked meat products.	Adverse effects of excessively consuming red meat have been demonstrated. Eat egg with yolk.	Consumption of fish lowers risk of CVDs, stroke (B), cognitive decline and macular degeneration (C). Excessive intake of meat increases all-cause mortality in men and risk of type 2 diabetes, colorectal cancer (B), and obesity (C). Higher meat intake lowers risk of iron deficiency anemia (C).
Cooking Oils Sodium Added Sugars Alcohol	10 10 5 5	Limit salt, cooking oil, added sugar, and alcohol. Limit cooking oils intake to 25–30g/day. Consume salt less than 6 g/day, and consume sodium less than 1500 mg/day. Limit intake of added sugars to less than 50 g/day. Children, adolescents, pregnant and lactating women should not consume alcohol; men (women) should limit alcohol intake to less than 25 g (15 g).	Cooking oils include plant oil and animal fat. The sodium component of the CHEI also includes sodium in sodium-rich foods (sodium content more than 500 mg/100 g).	Excessive consumption of any kind of fat increases energy intake and the risk of obesity (A). High consumption of sodium increases risk of hypertension (A), CVDs (C), stroke (B), gastric cancer (B). Overconsumption of added sugar increases risk of dental caries (B), weight gain (C), and hyperlipidemia (C). Excessive intake of alcohol rises risk of liver injury (A), gout (A), colorectal cancer (B), breast cancer (B), CVDs (B), and fetal alcohol syndrome (A).

**Table 2:** Recommended amounts of food groups, expressed in standard portion (SP) per 1000 kcal *.

Food Group	Standard Portion per 1000 kcal										
Calorie Level (kcal)	1000	1200	1400	1600	1800	2000	2200	2400	2600	2800	3000
Total Grains	1.7	1.7	2.1	2.5	2.5	2.5	2.5	2.5	2.7	2.7	2.7
Whole Grains and Mix Beans				0.6	0.8	1.0	1.1	1.3			
Tubers				0.3	0.3	0.4	0.3	0.4	0.5	0.4	0.4
Total Vegetables	2.0	2.1	2.1	1.9	2.2	2.3	2.0	2.1	1.9	1.8	2.0
Dark Vegetables	1.0	1.0	1.1	0.9	1.1	1.1	1.0	1.0	1.0	0.9	1.0
Fruits	1.5	1.3	1.1	1.3	1.1	1.5	1.4	1.5	1.3	1.4	1.3
Dairy	2.0	1.7	1.0	0.8	0.7	0.6	0.5	0.5	0.5	0.4	0.4
Soybeans	0.3	0.6	0.5	0.5	0.4	0.4	0.6	0.5	0.5	0.4	0.4
Seeds and Nuts				0.6	0.6	0.5	0.5	0.4	0.4	0.4	0.3
Fish and Seafood	0.3	0.4	0.6	0.6	0.6	0.6	0.8	0.7	0.6	0.8	0.9
Meat and Poultry	0.3	0.5	0.6	0.6	0.6	0.6	0.8	0.7	0.6	0.8	0.7
Eggs	0.4	0.5	0.4	0.6	0.5	0.6	0.5	0.5	0.4	0.4	0.4
Cooking Oils (g/1000 kcal)	15.0	16.7	14.3	15.6	13.9	12.5	11.4	12.5	11.5	10.7	11.7

* Specific recommended amounts for Whole Grains and Mixed Beans at 1000–1400 and 2600–3000 calories, for Tubers at 1000–1400 calories, and for Seeds and Nuts at 1000–1400 calories are not given by the Dietary Guideline for Chinese-2016, and they should be appropriate amounts.

**Table 3:** Chinese Healthy Eating Index (CHEI) components and standard for scoring.

Component	Score		
	0	5	10
Adequacy			
Total Grains	0 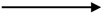	≥2.5 SP/1000 kcal	
Whole Grains and Mixed Beans	0 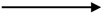	≥0.6 SP/1000 kcal	
Tubers	0 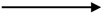	≥0.3 SP/1000 kcal	
Total Vegetables	0 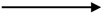	≥1.9 SP/1000 kcal	
Dark Vegetables	0 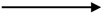	≥0.9 SP/1000 kcal	
Fruits	0 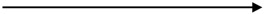		≥1.1 SP/1000 kcal
Dairy	0 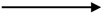	≥0.5 SP/1000 kcal	
Soybeans	0 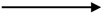	≥0.4 SP/1000 kcal	
Fish and Seafood	0 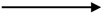	≥0.6 SP/1000 kcal	
Poultry	0 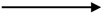	≥0.3 SP/1000 kcal	
Eggs	0 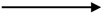	≥0.5 SP/1000 kcal	
Seeds and Nuts	0 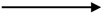	≥0.4 SP/1000 kcal	
Limitation			
Red Meat	≥3.5 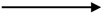	≤0.4 SP//1000 kcal	
Cooking Oils	≥32.6 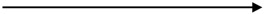		≤15.6 g/1000 kcal
Sodium	≥3608 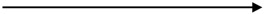		≤1000 mg/1000 kcal
Added Sugars	≥20% 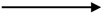	≤10% of energy	
Alcohol	≥25 g (men)/15 g (women) 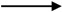	≤60 g (men)/40 g (women)	

**Table 4:** Characteristics of participants in tertiles ^a^ of CHEI score.

Characteristics	CHEI Tertiles				
	Total (*n* = 14,584)	Low (*n* = 4861)	Intermediate (*n* = 4862)	High (*n* = 4861)	*p* for Trend
CHEI Score	52.4 ± 10.9	40.6 ± 5.6	52.2 ± 2.7	64.3 ± 6.0	
Age group in years (%)					0.114
2–17	14.5	17.4	13.4	12.7	
18–59	61.0	53.9	63.8	65.3	
≥60	24.5	28.7	22.8	22.0	
Female sex (%)	52.0	52.7	49.6	53.7	0.330
Current smoking(%)	17.9	19.2	17.5	16.9	0.029
Living alone (%)	14.1	15.9	13.6	12.9	<0.001
Urbanization (%)					<0.001
Urban	24.3	13.6	18.5	40.7	
Suburban	33.2	33.3	33.8	32.4	
Rural	42.5	53.1	47.7	26.9	
Education level (%)					
Low	42.2	55.3	42.6	28.8	<0.001
Medium	47.3	39.4	49.0	53.4	
High	10.5	5.3	8.4	17.8	
Occupation (employed %)	49.4	45.1	52.9	50.2	<0.001
BMI					<0.001
Under weight	14.0	17.5	13.5	11.1	
Normal	47.5	46.1	48.5	47.9	
Over weight	28.8	26.8	28.0	31.4	
Obesity	9.7	9.6	10.0	9.6	

^a^ Tertiles of CHEI score were based on the data of 24-h recall over the three consecutive days. Body mass index (BMI).

**Table 5:** Estimated mean and percentiles of the CHEI in 14,584 participants from CHNS-2011, based on three days of dietary consumption.

Percentile										
CHEI Score	Mean	1st	5th	10th	25th	50th	75th	90th	95th	99th
Total	52.4	27.6	34.6	38.7	45.0	52.2	59.6	66.5	70.9	78.3
Men	52.2	27.9	35.2	39.1	45.2	52.1	59.2	65.6	69.8	76.6
Women	52.5	27.5	34.1	38.2	44.7	52.3	60.0	67.5	71.6	78.9
2–17	50.4	22.9	31.1	35.2	42.3	50.9	58.5	65.2	69.4	76.9
18–59	53.2	29.1	36.2	39.8	46.3	53.1	60.1	67.0	70.9	77.8
60+	51.4	27.6	33.8	37.9	43.6	50.6	58.6	66.1	71.5	79.8

**Table 6:** Linear regression models for total CHEI, classified by associated factors.

Variables	CHEI Score	Univariate Model		Final Multivariate Model ^a^	
	Mean (SD)	Coefficient (95%CI)	*p*-Value	Coefficient (95%CI)	*p*-Value
Sex					
Men	52.2 (10.4)	Reference		Reference	
Women	52.5 (11.3)	0.27 (−0.08, 0.63)	0.130	0.36 (−0.02, 0.73)	0.070
Age groups					
18–59	53.2 (10.5)	Reference		Reference	
2–17	50.4 (11.6)	−2.88 (−3.40, −2.37)	<0.001	−0.32 (−2.09, 1.44)	0.719
≥60	51.4 (10.9)	−1.86 (−2.28, −1.44)	<0.001	−0.93 (−1.44, −0.43)	<0.001
Education level					
Low	49.3 (10.3)	Reference		Reference	
Medium	53.8 (10.5)	4.50 (4.14, 4.86)	<0.001	2.66 (2.25,3.06)	<0.001
High	58.4 (11.1)	9.06 (8.47, 9.65)	<0.001	5.13 (4.47, 5.78)	<0.001
Smoking					
Non-smoker	52.5 (10.9)	Reference		Reference	
Former smoker	54.4 (11.1)	1.96 (0.76, 3.16)	0.001	0.30 (−0.85, 1.44)	0.609
Current smoker	51.8 (10.5)	−0.72 (−1.19, −0.26)	0.002	−1.29(−1.78, −0.80)	<0.001
Living alone					
Yes	51.3 (10.9)	Reference		Reference	
No	52.6 (10.9)	1.28 (0.78, 1.79)	<0.001	1.75 (1.24, 2.24)	<0.001
Urbanization					
Rural	49.3 (9.5)	Reference		Reference	
Suburban	52.1 (10.5)	2.79 (2.40, 3.18)	<0.001	1.95 (1.55,2.35)	<0.001
Urban	58.1 (11.4)	8.77 (8.34, 9.19)	<0.001	6.83 (6.35, 7.31)	<0.001
Occupation					
Employed	52.9 (10.2)	Reference		Reference	
Unemployed	50.1 (10.4)	−2.77 (−3.24, −2.29)	<0.001	−1.69 (−2.17, −1.22)	<0.001
Retired	55.0 (11.7)	2.14 (1.65, 2.62)	<0.001	0.96 (0.38, 1.55)	0.001
At school	52.1 (11.1)	−0.72 (−1.30, −0.14)	0.015	0.69 (−1.02, 2.40)	0.428
Dropouts	46.5 (12.2)	−6.38 (−7.27, −5.48)	<0.001	−3.73 (−5.67, −1.79)	<0.001
BMI					
Normal	52.6 (10.6)	Reference		Reference	
Underweight	49.9 (11.3)	−2.73 (−3.27, −2.20)	<0.001	−0.73 (−1.36, −0.09)	0.024
Overweight	53.2 (10.9)	0.59 (0.18, 1.01)	0.005	0.27 (−0.12, 0.66)	0.176
Obesity	52.4 (10.9)	−0.12 (−0.74, 0.49)	0.693	0.05 (−0.53, 0.62)	0.875

^a^ Final multivariate model shows the relationship between the CHEI scores and the potential impact factors; all factors listed, except sex, are significantly associated with overall diet quality. Standard deviation (SD).

## Supplementary Materials

The following are available online at www.mdpi.com/2072-6643/9/9/977/s1, Figure S1: Current nutritional status of China, Table S1: Standard portion (SP) size for ten major food groups based on the DGC-2016, Table S2: Recommended amounts of food groups, expressed in gram * (a) and expressed in standard portion (SP) size * (b).
