# Supplementary material for: The Development of a Chinese Healthy Eating Index and Its Application in the General Population
Source: Nutrients. 2017 Sep 5;9(9):977. doi: 10.3390/nu9090977 (PMC5622737; doi:10.3390/nu9090977)
Supplement: Supplementary file 1 [file nutrients-09-00977-s001.zip › nutrients-212692-supplementary.pdf]

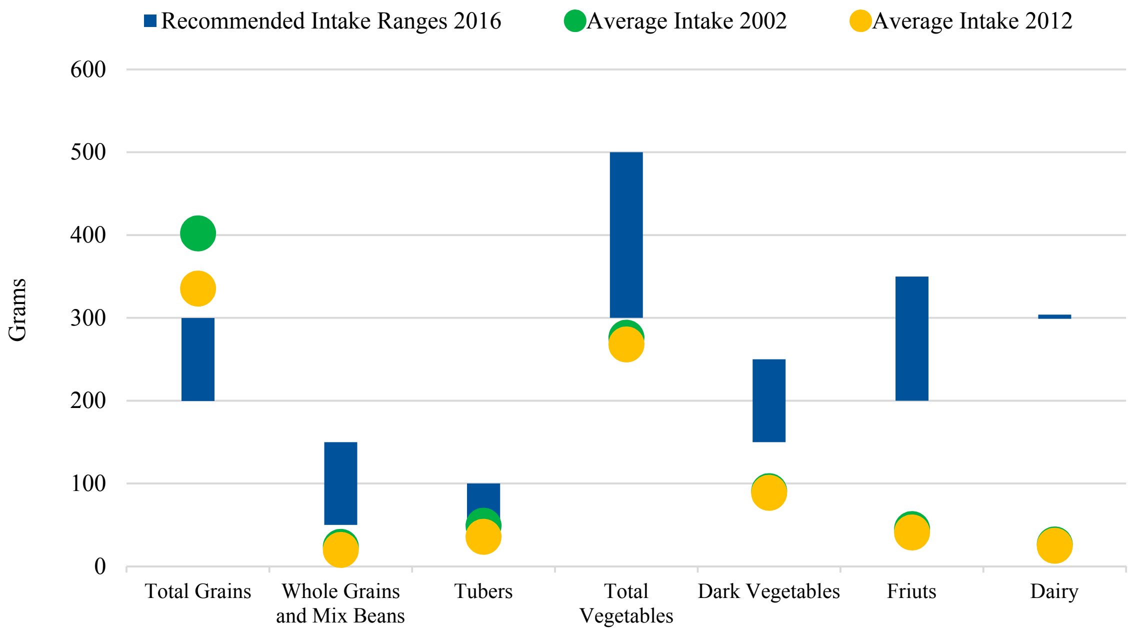
Recommended Intake Ranges 2016
Average Intake 2002
Average Intake 2012
Grams
600
500
400
300
200
100
0
Total Grains
Whole Grains and Mix Beans
Tubers
Total Vegetables
Dark Vegetables
Friuts
Dairy

(**a**)

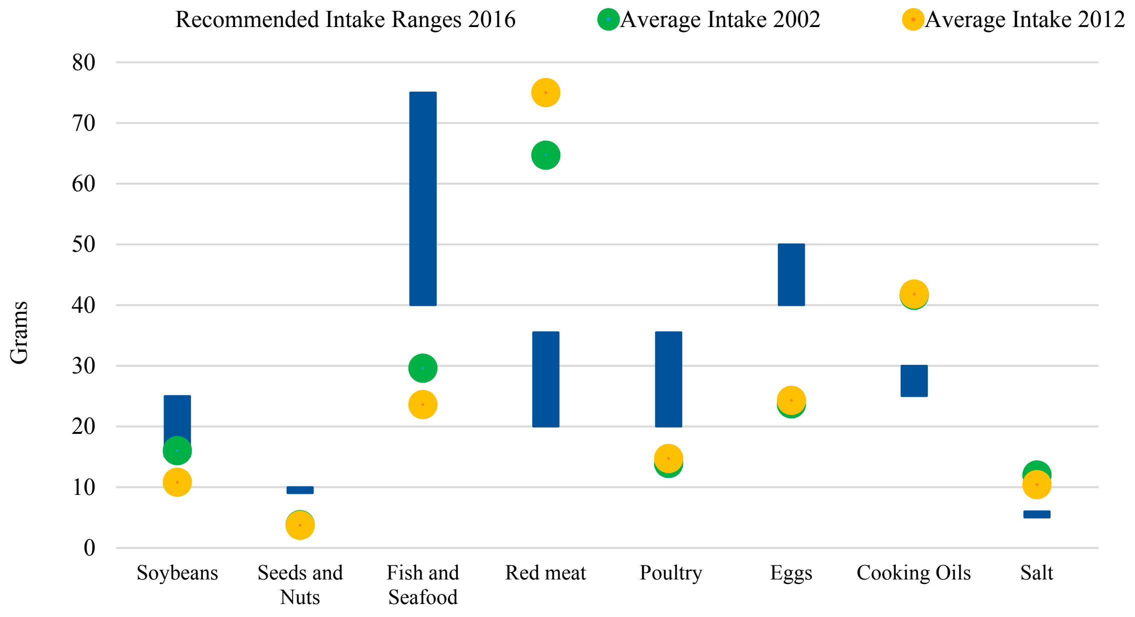

(**b**)

Figure S1. Current nutritional status of China. Added sugars were not included in Supplementary Figure S1a,b because the data sources were from the Chinese Nutrition and Health Surveillance 2002 and 2012, which did not incorporate the data on added sugars.

Table S1. Standard portion (SP) size for 10 major food groups based on the DGC-2016.

Food categories	Energy per SP (kcal)	Carbohydrate per SP (g)	Protein per SP (g)	Fat per SP (g)	Examples of one SP
Grains	160–180	35–40	4–7	0–2	wheat flour 50 g, wheat bun 70–80 g rice (raw) 50 g, rice (steamed) 100–120 g
Tubers	80–90	18–22	0–4	0–1	potato 100 g, sweet potato 80 g
Vegetables	15–35	3–7	1–3	0–1	spinach 100 g, snow pea 80 g
Fruits	40–55	10–15	0–1	0–1	apple 100 g, Chinese date 25 g, pear 100 g, persimmon 65 g
Dairy					
(whole fat% ≈ 3%)	110	10–20	5–6	5–7	milk 200 mL, cheese 20~25 g, milk powder 20~30 g
(low fat% < 0.5%)	55			2–3	
Soybeans and products	65–80	4–8	6–8	3–8	soybean 20 g, soybean curd 60 g soybean curd slab 45 g soybean milk 360~380 mL
Seeds and Nuts	40–55	1–10	1–3	2–4	peanut 10 g, chestnut 25 g lotus seed 20 g
Fish	50–60	0–5	6–10	1–4	grass carp 50 g, shrimp 50 g,
Seafood	35–50				hairtail 45 g, clam 45 g
Meat and Poultry					
(fat % < 10%)	40–50	0–5	5–10	1–5	lean meat 50 g, streaky pork 25 g
(fat% >= 10%)	10–25		2–8	5–8	
Eggs	40–50	1–3	7	5–10	Eggs 50 g

Table S2. Recommended amounts of food groups, expressed in grams* (**a**) and expressed in standard portion (SP) size* (**b**).

a.

Food Group (g)											
Calorie level (kcal)	1000	1200	1400	1600	1800	2000	2200	2400	2600	2800	3000
Total Grains	85	100	150	200	225	250	275	300	350	375	400
Whole Grains and Mixed Beans				50	75	100	125	150			
Tubers				50	50	75	75	100	125	125	125
Vegetables	200	250	300	300	400	450	450	500	500	500	600
Dark Vegetables	100	125	150	150	200	225	225	250	250	250	300
Fruits	150	150	150	200	200	300	300	350	350	400	400
Dairy	500	500	350	300	300	300	300	300	300	300	300
Soybeans	5	15	15	15	15	15	25	25	25	25	25
Seeds and Nuts				10	10	10	10	10	10	10	10
Fish and Seafood	15	20	40	40	50	50	75	75	75	100	125
Meat and Poultry	15	25	40	40	50	50	75	75	75	100	100
Eggs	20	25	25	40	40	50	50	50	50	50	50
Cooking Oils	15	20	20	25	25	25	25	30	30	30	35

b.

Food Group (SP)											
Calorie level (kcal)	1000	1200	1400	1600	1800	2000	2200	2400	2600	2800	3000
Total Grains	1.7	2.0	3.0	4.0	4.5	5.0	5.5	6.0	7.0	7.5	8.0
Whole Grains and Mixed Beans				1.0	1.5	2.0	2.5	3.0			
Tubers				0.5	0.5	0.8	0.8	1.0	1.3	1.3	1.3
Vegetables	2.0	2.5	3.0	3.0	4.0	4.5	4.5	5.0	5.0	5.0	6.0
Dark Vegetables	1.0	1.3	1.5	1.5	2.0	2.3	2.3	2.5	2.5	2.5	3.0
Fruits	1.5	1.5	1.5	2.0	2.0	3.0	3.0	3.5	3.5	4.0	4.0
Dairy	2.0	2.0	1.4	1.2	1.2	1.2	1.2	1.2	1.2	1.2	1.2
Soybeans	0.3	0.8	0.8	0.8	0.8	0.8	1.3	1.3	1.3	1.3	1.3
Seeds and Nuts				1.0	1.0	1.0	1.0	1.0	1.0	1.0	1.0

Fish and Seafood	0.3	0.4	0.9	0.9	1.1	1.1	1.7	1.7	1.7	2.2	2.8
Meat and Poultry	0.3	0.6	0.9	0.9	1.1	1.1	1.7	1.7	1.7	2.2	2.2
Eggs	0.4	0.6	0.6	0.9	0.9	1.1	1.1	1.1	1.1	1.1	1.1
Cooking Oils (g)	15.0	20.0	20.0	25.0	25.0	25.0	25.0	30.0	30.0	30.0	35.0

* Specific recommended amounts for Whole Grains and Mixed Beans at 1000–1400 and 2600–3000 calories, for Tubers at 1000–1400 calories, and for Seeds and Nuts at 1000–1400 calories are not given by the Dietary Guideline for Chinese-2016, and they should be appropriate amounts.
